# The Functional Haplotypes of* CHRM3* Modulate mRNA Expression and Associate with Bladder Cancer among a Chinese Han Population in Kaohsiung City

**DOI:** 10.1155/2016/4052846

**Published:** 2016-12-07

**Authors:** Chiang-Ting Wang, Tsung-Ming Chen, Chien-Tai Mei, Chun-Feng Chang, Li-Lian Liu, Kuo-Hsun Chiu, Tsung-Meng Wu, Yu-Ching Lan, Wen-Sheng Liu, Ya-Huey Chen, Yi-Mei Joy Lin

**Affiliations:** ^1^Division of Urology, Department of Medicine, Kaohsiung Armed Forces General Hospital, Kaohsiung, Taiwan; ^2^Department and Graduate Institute of Aquaculture, National Kaohsiung Marine University, Kaohsiung, Taiwan; ^3^Department of Oceanography, National Sun Yat-sen University, Kaohsiung 804, Taiwan; ^4^Department of Aquaculture, National Pingtung University of Science and Technology, Pingtung, Taiwan; ^5^Department of Health Risk Management, China Medical University, Taichung, Taiwan; ^6^Asia-Pacific Biotech Developing Inc., Kaohsiung, Taiwan; ^7^Graduate Institute of Biomedical Science, China Medical University, Taichung, Taiwan; ^8^Center for Molecular Medicine, China Medical University Hospital, Taichung, Taiwan; ^9^Cancer Biology and Drug Discovery Ph.D. Program, College of Medicine, China Medical University, Taichung, Taiwan; ^10^Institute of Biomedical Sciences, National Chung Hsing University, Taichung, Taiwan

## Abstract

Bladder cancer is one of the major cancer types and both environmental factors and genetic background play important roles in its pathology. Kaohsiung is a high industrialized city in Taiwan, and here we focused on this region to evaluate the genetic effects on bladder cancer. Muscarinic acetylcholine receptor M3 (CHRM3) was reported as a key receptor in different cancer types.* CHRM3 *is located at 1q42-43 which was reported to associate with bladder cancer. Our study attempted to delineate whether genetic variants of* CHRM3* contribute to bladder cancer in Chinese Han population in south Taiwan. Five selected SNPs (rs2165870, rs10802789, rs685550, rs7520974, and rs3738435) were genotyped for 30 bladder cancer patients and 60 control individuals and genetic association studies were performed. Five haplotypes (GTTAT, ATTGT, GCTAC, ACTAC, and ACCAC) were found significantly associated with low* CHRM3* mRNA level and contributed to increased susceptibility of bladder cancer in Kaohsiung city after rigid 10000 consecutive permutation tests. To our knowledge, this is the first genetic association study that reveals the genetic contribution of* CHRM3 *gene in bladder cancer etiology.

## 1. Introduction

Bladder cancer is one of the major cancers worldwide. In 2013, bladder cancer is the eighth and tenth most frequently diagnosed cancer among men and women [1]. About 72571 new bladder cancer cases and 15210 cancer deaths were also reported in 2013 [1]. Although the etiology of cancer has not been fully established, it is mostly accepted as a multifactorial disease involving both environmental and genetic factors [2, 3]. The related risk factors for bladder cancer included interactions between genetic background and environmental factors, for example, tobacco smoking, industrial chemicals (aromatic amine) exposure, chronic infection with* Schistosoma* species, oxidants, and highly reactive free radicals [3].

Geographically, Kaohsiung is located in south Taiwan at 22.63 degrees north latitude and 120.30 degrees east longitude. The area of Kaohsiung city is 2952 square kilometers containing 1387931 people and is the second most populated city in Taiwan. Kaohsiung is a high industrialized city in Taiwan containing at least 8 industrial areas and 6306 plants in operation (http://erdb.epa.gov.tw/ERDBIndex.aspx; Supplemental Table  1 in Supplementary Material available online at http://dx.doi.org/10.1155/2016/4052846). Many types of industries were reported in Kaohsiung city, including semiconductor, optoelectronics, electroplating, steel, shipbuilding, leather, and dyeing. Thousands of industrial wastes were discharged from these plants every day. Therefore, people living in Kaohsiung are under a high exposure level to industrial pollution which has been reported as a key environmental factor for many cancer types, especially cancers in urinary system [4]. As Taiwan Cancer Registry Annual Report 2012 [5], the incidence of bladder cancer in south Taiwan was 9.73 per 100,000 people, which was the highest area in west Taiwan. Notably, most of the new cases of bladder cancer were reported in Kaohsiung city. Therefore, Kaohsiung city is suitable for evaluating the pathological factor of bladder cancer.

Genetic predisposition is an important risk factor related to bladder cancer. Recently, 12 genes were identified to associate with bladder cancer at a genome-wide significant level in European populations from six genome-wide association studies (GWAS) [6–11]. Among these 12 genes, 12 independent single nucleotide polymorphisms (SNPs) have been evaluated and six genes (seven SNPs) showed association with bladder cancer in Chinese populations, including* CLPTM1L* (rs401681) [12],* TP63* (rs710521) [13],* TACC3* (rs798766) [14, 15],* TERT* (rs2736100 and rs2736098) [12, 15],* PSCA* (rs2294008) [15, 16], and* MYC* (rs9642880) [15, 17]. However, whether other genes are also playing pathological roles in bladder cancer in the Chinese population remains unknown.

Prior studies revealed that G protein-coupled receptors (GPCRs) pathways regulated kidney and bladder cancer cell lines migration and invasion [18]. GPCRs have enormous potential in biomedical research and drug development resulting in their features and functions to serve as therapeutic targets [19]. Acetylcholine, the first identified neurotransmitter, has been shown to exert lots of its physiological action of GPCRs and modulate neuronal differentiation during early development. Both muscarinic and nicotinic acetylcholine receptors (AChRs) regulate physiological responses widely, including apoptosis, cellular proliferation, and neuronal differentiation [20]. However, the role of muscarinic signaling in neoplasia has exhibited relatively scant attention. Muscarinic acetylcholine receptors are similar to other G protein coupled receptors and have seven transmembrane helical domains connected by three extracellular and three intracellular loops. Muscarinic AChR (CHRM) family comprising five subtypes, M1R to M5R (gene names,* CHRM1*-*CHRM5*), regulates cell function via different postreceptor signaling pathways [21]. Robust evidence supporting activation of these receptors plays a critical role in regulation broad-range cancers [22]. Activation of M3R stimulated MEK/ERK signaling to promote cancer cell proliferation in several cancers including brain, breast, colon, and prostate cancers [22].

M3R is the major receptor mediating urinary bladder contraction upon micturition [23]. In normal human urinary bladder, the* CHRM3* mRNA expression in bladder areas related to micturition, such as the bladder dome, neck, and trigone [24]. Genetically,* CHRM3* gene was mapped to chromosome 1q43. Mutation of* CHRM3 *showed defects in urinary system, including malformed bladders [25]. Vauhkonen et al. showed that duplication of 1q42-43 was identified in bladder cancer by array CGH analysis [26]. These studies demonstrated that* CHRM3 *plays a vital role in bladder development and suggested that it may also have pathophysiological role in bladder cancer. However, no solid evidence showed the genetic effect of* CHRM3* gene polymorphism on bladder cancer. Thus, we attempted to delineate the genetic association of* CHRM3* genotypes and bladder cancer. It is valuable to realize the putative efficiencies in clinical application.

## 2. Materials and Methods

### 2.1. Sample Collection

For association analysis, 30 individuals with bladder cancer and 60 noncancer control individuals were recruited in this study (proofed by the institutional review board of Kaohsiung Armed Forces General Hospital). All of them were interviewed and diagnosed by Dr. Chiang-Ting Wang; basic characteristics of both groups were summarized in Table 1. Three milliliter blood samples were collected into EDTA-anticoagulants tubes and their genomic DNA was isolated from blood samples using the DNeasy™ kit (QIAamp DNA Blood Mini kit, Qiagen, Valencia, Germany). Briefly, the blood was digested with 0.5 mg/mL proteinase K in 400 *μ*L cell-lysis solution at 55°C for 24 h until the blood was completely lysed. After adding 200 *μ*L absolute ethanol to the reagents, the mixture was transferred into the DNeasy minicolumn and centrifuged at 8,000 rpm for 1 min. The DNeasy minicolumn was washed with 500 *μ*L washing buffer and centrifuged for 1 min at 8,000 rpm. Finally, the DNA was eluted in a clean 1.5 mL microcentrifuge tube.

### 2.2. Tumor Tissue Samples Collection

Thirty bladder cancer tissues of patients and fifty-nine control bladder samples were collected. All samples were frozen in liquid nitrogen immediately after surgical remove and then stored in −70°C.

### 2.3. Polymorphism Selection and SNPs Genotyping

Five SNPs of* CHRM3* gene were selected based on genetic information in HapMap (http://www.hapmap.org/), depending on their genomic distribution and allele frequencies in our population. Allele-specific polymerase chain reaction (AS-PCR) analysis was performed with allele-specific primer sets (Table 2) to genotype alleles of* CHRM3* (rs2165870, rs10802789, rs685550, rs7520974, and rs3738435). Briefly, forward primers were designed specifically complementary to each SNP allele and two PCR reactions were conducted with these two specific primer sets in parallel and then detected the allele by using Mycycle™ thermal cycler (Bio-RAD, Hercules, CA, USA). PCR reagents contained 1x PCR Buffer, 1 U of Taq DNA polymerase (TakaRa, Shuzo, Kyoto, Japan), 100 ng of each primer, 200 *μ*mol/L dNTP, and 10 ng of genomic DNA. DNA amplification was through denaturation at 94°C for 5 min; 30 cycles of denaturation at 94°C for 30 s, annealing at 55°C for 30 s, extension at 72°C for 30 s, and final extension at 72°C for 10 min. PCR products were analyzed by 2% agarose gel and also further confirmed via Sanger DNA sequencing.

### 2.4. Reverse Transcription-Polymerase Chain Reaction (RT-PCR)

Total RNA (1 *μ*g) was used as templates to generate complementary DNA (cDNA) by using the Moloney Murine Leukemia Virus reverse-transcriptase kit according to the manufacturer's instructions (Life Technologies Inc., Paisley, UK). The expression level of* CHRM3* transcript was further quantified by specific primers listed in Table 2. PCR amplifications of* CHRM3* transcript were through denaturation at 94°C for 2 min; 28 cycles of denaturation at 94°C for 1 min, annealing at 55°C for 30 sec, extension at 72°C for 1 min, and final extension at 72°C for 10 min. RT-qPCR of* CHRM3 *and *β*-actin used TaqMan assays (Applied Biosystems) in a real-time PCR system (StepOne™; Applied Biosystems). The levels of* CHRM3* mRNA were measured using the 2^−ΔΔCt^ method and normalized to the expression levels of *β*-actin.

### 2.5. Statistical Analysis

Hardy-Weinberg equilibrium (HWE) was tested by online HWE program (http://linkage.rockefeller.edu/ott/linkutil.htm), allele and genotype frequencies examined by Fisher's exact (two-tailed) test, using SPSS version 10.0 software (SPSS for Windows Inc., Chicago, IL). Haplotype construction and the differences in haplotype distributions between patients and controls were analyzed using SNPAlyze (SNPAlyze 4.1; Dynacom Co. Ltd., Kanagawa, Japan), *p* value < 0.05 constituting statistical significance. Odds ratios with 95% confidence intervals were also computed. Additional rigid 10000 consecutive permutation tests for haplotype association were calculated using SAS 8.0 program (SAS, Cary, NC, USA). Powers were estimated using Power Analysis and Sample Size (PASS) 2005 software (NCSS, Kaysville, UT, USA).

Results of RT-qPCR were analyzed using GraphPad Prism 5.0 (GraphPad Software, San Diego, CA), all data were represented as mean ± standard deviation (SD), and a *p* value < 0.05 was considered as a significant difference.

## 3. Results and Discussion

Urothelial bladder cancer is a multifactorial disease and its etiology is highly complex. Studies showed that bladder cancer is associated with infection, injury, chronic inflammation, tissue repair, and genetics factors [3, 27]. Current theory suggests that using association analysis will provide acceptable power to detect genetic effects on complex diseases [28]. Many risk-associated genetic variants were linked to contribute a subtle effect to complex diseases, such as cancer, by case-control association studies [28]. Although CHRM3 cholinergic receptor has been reported to promote cancer cell proliferation via stimulating MEK/ERK signaling in several different cancers [22], its role in the bladder cancer have not been estimated yet. Industrial exposure is a very important environmental factor for cancers in urinary system [29]. Kaohsiung city, with high plant density, especially lots of high-polluting industries, such as petroleum, petrochemical, dyeing, steel, and iron industries [8, 30], should be chosen to conduct study on the association between environmental or genetic factors and urinary cancer. In this study, we analyzed association of bladder cancer with* CHRM3* polymorphism of Taiwanese population in Kaohsiung city, an industrial city with high exposure level of industrial pollution, to evaluate the* CHRM3* genetic effect on etiology of bladder cancer.

Bladder cancer patients and volunteer controls who joined this study were characterized by age, body mass index (BMI), male-to-female ratio, smoking status, alcohol drinking status, and urinalysis. Data were summarized in Table 1. With respect to gender ratio, smoking status, alcohol drinking and uric acid, and the control and bladder cancer groups showed no difference in these analyses. According to the Taiwan Cancer Registry Annual Report 2012, patients aged over 50 years account for over 93% of bladder cancer patients. Although this study does not match the cases and controls for age, the average age (53 yrs) of our control groups is slightly older than 50 years.

**Figure 1:**
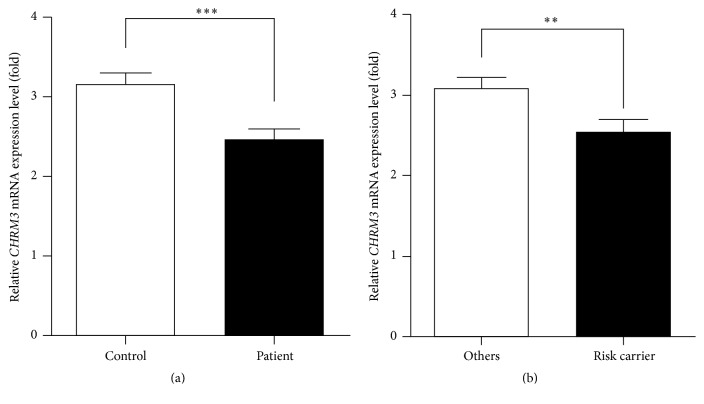
*CHRM3 *mRNA level is associated with bladder cancer and risk haplotype carrier groups. (a) Bar graph represented the relative* CHRM3* mRNA level quantified by real-time qPCR in bladder cancer group and control.* CHRM3* mRNA level was decreased to 0.78-fold (*p* < 0.0001) in bladder cancer group compared to control. The bars represent the mean ± SD. ^*∗∗∗*^ *p* < 0.001. (b) Bar graph represented the relative* CHRM3* mRNA level quantified by real-time qPCR in risk carrier and others group.* CHRM3* mRNA level was decreased to 0.82-fold (*p* = 0.0035) compared to others group. The bars represent the mean ± SD. ^*∗∗*^ *p* < 0.01.

To reveal the expression level of* CHRM3* mRNA in bladder tissue of patients and controls, the levels of* CHRM3* and *β*-actin mRNA on 29 patients and 59 controls were measured using real-time qPCR assay. Result showed that the relative level of* CHRM3* mRNA was decreased to 0.78-fold (*p* < 0.0001) in bladder cancer group (Figure 1(a)), implying that CHRM3 expression level might be associated with bladder cancer. To test the genetic association of the* CHRM3* polymorphism with bladder cancer, five selected SNPs were genotyped for all our participants. Genotypes of these five SNPs were further confirmed by Sanger DNA sequencing (Supplemental Figure  1). The average genotyping success rate was about 99%. General population-based genotypic and allelic distributions were similar to the data from the Single Nucleotide Polymorphism database (dbSNP) and international HapMap project (https://hapmap.ncbi.nlm.nih.gov/). All SNPs were analyzed to compare allelic and genotypic frequencies between bladder cancer group and control group using the Pearson *χ* ^2^ test. There were no significant differences of alleles or genotype distributions in single locus association (Table 3).

Haplotype-based association analysis comprising multiple SNPs can provide additional power for mapping disease genes and also provide insight on factors influencing the dependency among genetic markers and identifying cis-interactions between two or more variants. Therefore, we investigated the genetic associations in haplotype-based analysis for* CHRM3* gene with bladder cancer. Haplotype-based genetic association tests were done using the estimated haplotype frequencies. Although there was no single locus association between any* CHRM3* polymorphisms with bladder cancer, there was a significantly different distribution of several haplotypes between groups (Table 4). In addition, significant differences on some haplotypes were detected even after rigid 10000 consecutive permutation tests. The distribution of the most common haplotype in control groups, GTTAC, showed a significant protective effect (permutation *p* = 0.027; OR: 0.35, 95% CI: 0.136–0.903, power = 61% at *α* = 0.05). And 4 of the demonstrated 16 haplotypes only occurred in control groups (ACTGT, GCCAT, ATTAC, and ACCGC) and also showed protective effect for bladder cancer. On the other hand, haplotype GTTAT (*p* = 0.029; OR: 3; 95% CI: 1.079–8.344) and ATTGT (*p* = 0.036; OR: 3.292; 95% CI: 1.027–10.56) are associated with significantly risk effects. However, after rigid permutation analysis, haplotype ATTGT was not significantly associated. There are also three haplotypes only observed in bladder cancer patients (GCTAC, ACTAC, and ACCAC) and showed risk effect for bladder cancer. Significant differences of GCTAC were still detected after rigid consecutive permutation tests (permutation *p* = 0.044; power = 66% at *α* = 0.05). Significant differences were also assessed in overall haplotype frequency profiles (total *p* = 0.0019; global permutation *p* = 0.0008, power = 99% at *α* = 0.05).

Interestingly, we found that although the selected* CHRM3* gene polymorphisms were not significantly associated with bladder cancer on single locus level, the interaction-considered haplotype-based analysis showed that the* CHRM3* gene was significantly associated with bladder cancer. To reveal if these risk haplotype indeed has biological effect, we measure the mRNA expression level on risk haplotype carriers and noncarriers. SNPs located at regulatory regions were considered as functional variants for gene expression and contribute to the disease risk [31, 32]. All of the selected SNPs are located on 5′ introns and may play a role in regulating* CHRM3* gene expression [4]. Therefore, we hypothesized that the* CHRM3* gene expression level on risk haplotype carriers may differ compared to other individuals. To reveal our hypothesis, we performed cross-analysis of the* CHRM3 *mRNA qPCR data with risk haplotype analysis. Based on the haplotype data, all of the involved individuals were regrouped into risk haplotype carriers (GTTAT, ATTGT, GCTAC, ACTAC, and ACCAC; odds ratio > 1, 95% CI > 1, or only observed in bladder cancer patients) (*n* = 28) and others (*n* = 62), and then their* CHRM3 *mRNA expression level was compared. Surprisingly, the relative amount of* CHRM3 *mRNA was significantly reduced to 0.82-fold in the risk haplotype carriers group compared to others (*p* = 0.0035; Figure 1(b)). These data revealed that at least some of the selected SNPs have biological function and affect the* CHRM3* gene expression. To our knowledge, this is the first association study to reveal the contribution of the genetic effect of* CHRM3 *gene in bladder cancer, and to expose that these risk haplotypes not only are genetically associated but indeed correlate with altered CHRM3 gene expression level, at least among the studied Chinese Han population in Kaohsiung city.

Cholinergic receptors are generally classified into muscarinic or nicotinic categories depending on the binding affinity of two naturally occurring substances, muscarine and nicotine. G proteins regulate extensively biological processes by modulating the activity of adenylyl cyclase, phosphatidylinositol lipid turnover, and ion channels [33]. The G protein- and acetylcholine- (ACh-) binding site are located at intracellular loop and sixth transmembrane domain, respectively [22]. The CHRM family regulates cell function via different postreceptor signaling pathways [21]. The M1R, M3R, and M5R exhibit selectively G proteins of G_q/11  _ family, which activate phospholipase C-*β* to initiate the phosphatidylinositol trisphosphate signaling cascade. Activation of these 3 receptors causes phospholipid turnover and alters cell calcium concentration. The M2R and M4R preferentially couple to G_i/o_ type G proteins that inhibit adenylyl cyclase activity [34]. Activating these two receptors results in suppression of adenylyl cyclase and attenuated levels of cAMP.

Muscarinic receptors are well defined and long-established as apparatus in neuronal system [35]. Likewise, muscarinic receptors are expressed in various organ systems including gastrointestinal tract, brain, eye, heart, lung bronchioles, urinary bladder, and uterus [36]. The pathological roles of muscarinic receptors have also been reported in 1991. Muscarinic receptors are conditional oncogenes by activating downstream phosphatidylinositol hydrolysis when they are expressed in cells capable of proliferation [37]. Muscarinic receptor was also linked to brain, breast, colon, lung, prostate cancer, and melanoma [22]. Robust evidence supports the idea that activation of these receptors plays a critical role in regulation broad-range cancers [22]. It has been found that CHRM3 receptors are abundantly expressed on 60% of studied colon cancer cell lines (6/10) [38]. In one small study, five of the eight (62%) showed an up to 8-fold expression level of* CHRM3* mRNA compared with the matched normal tissue [39]. Activation of M3R stimulated MEK/ERK signaling to promote cancer cell proliferation in several cancers including brain, breast, colon, and prostate cancers [22]. Postmuscarinic receptor signaling cascade included NF-*κ*B, AKT, and cyclin D1 signaling pathways resulting in augment cell survival in diverse cancers. These pathways raise gene transcription favoring cell proliferation and survival, both hallmarks of neoplasia. Furthermore,* CHRM3* knockout mice were shown to attenuate intestinal tumor number and size, revealing an oncogenic role of* CHRM3* in cancer progression [40]. These studies suggested a functional role of* CHRM3* in cancer and further support the result from our genetic study. Although studies showed CHRM3 mRNA are overexpressed in colon cancer and activation of M3R stimulated MEK/ERK signaling to promote cancer cell proliferation, less study was performed to reveal the role of CHRM3 in bladder cancer. The rodent cancer model showed that increased fecal bile acids promote colon dysplasia through interacting with muscarinic receptors [41]. Therefore, the overreactive signaling of muscarinic receptors may be caused by increasing the amount of the receptor or its ligands [42]. Our data showed a minor reduction of* CHRM3* mRNA in risk haplotype carriers group and bladder cancer samples, distinct from finding on colon cancer studies (Figure 1(b)). The reason may result from different pathological mechanism, different clinicopathological features of the studied patients, tissue-specific expression of* CHRM3* mRNA, or small sample size. It is not uncommon that single gene has multifaceted role in different cancer types. For example, PRL-3 gene plays a tumor suppressor role in lung cancer, distinct from observation on colon and gastric cancers [43]. Recently, one study revealed that CHRM3 expression level was correlated with some clinicopathological features, such as FIGO stage, vascular invasion, and lymphatic metastasis [44]. Majority of our cancer patients belong to the FIGO stage I with no regional lymph node and distant metastasis that should fit into the low expression group. However, the analyzed cell lines and cancer patients in colon cancer studies are more clinical heterogeneity and may lead to different CHRM3 expression patterns. One of the other possibilities is the cell type-specific regulation of* CHRM3* mRNA expression. The cell type of bladder cancer samples in our study is all urothelial carcinoma, also known as transitional cell carcinoma (TCC), and the most common type of colon cancer is adenocarcinoma. Gene regulation machinery and their role on the carcinogenesis could be different in different cell types. And the actual roles of CHRM-3 in bladder cancer etiology are needed to be clarified.

## 4. Conclusions

Evidence from our genetic and functional studies suggested that some risk haplotypes were significantly associated with low* CHRM3* mRNA level and contributed to increased susceptibility of bladder cancer in Kaohsiung city. In conclusion, results from other research groups and ours confirm the importance of muscarinic signaling in bladder cancer, which further suggests that intervention of muscarinic signaling may provide novel therapeutic or prognostic strategies for this disease. Although the association of bladder cancer and* CHRM3* is significant, we could not completely exclude a possibility of false-positives because of small sample size or other factors (e.g., confounders with other medical conditions and adjustment of other risk factors). However, we have provided additional power estimation and rigid permutation test to support our conclusion. Moreover, besides the SNPs explored in this study, there may have been other polymorphisms associated with bladder cancer; a systematic association study with a large sample size is considerable in the following study.

## Supplementary Material

The Supplementary Material included one supplementary table and one supplementary figure as follows. Supplementary Table1: Summary of plants and populations on different areas in Taiwan, 2012. Supplementary Figure S1: Sequencing results of CHRM3 SNPs.

## Figures and Tables

**Table 1:** Demographic data of individuals collected in this study.

	Patients (*n* = 31)	Controls (*n* = 60)	*p*value^a^
Age (years)	70.47 ± 12.6	53.02 ± 13.66	0.001^*∗*^
Gender (M/F)^b^	25/6	48/12	0.942
Smoking status (Y/N)^c^	7/12	17/31	0.913
Alcohol drinking (Y/N)^c^	4/15	11/36	0.836
BMI	25.62 ± 5.08	24.95 ± 4.24	0.506
Uric acid (mg/day)	6.72 ± 2.39	6.97 ± 2.76	0.669

Data are presented as mean ± standard deviation.

^a^ *Chi*-squared tests and student's *t*-tests were used to perform statistical analysis of differences between the groups. ^*∗*^ *p* < 0.05.

^b^F, female; M, male.

^c^Y, yes; N, no; information of some individuals is unavailable.

**Table 2:** Primers utilized in this study.

SNP	Primers, 5′-3′	Product Size (bp)	Function
rs2165870 (A/G)	F: TCCTAGGGCTCTGAAGAATTA	383	PCR
	R: TTCAATTGACATTGCTCAGA		
	FA: TGCATCTGTTTACAGCCTTA	306	Genotyping
	FG: TGCATCTGTTTACAGCCTTG		

rs10802789 (C/T)	F: CTCGAAGTTAGGCGAGATTT	397	PCR
	R: GTGTGACACGTGTAGGTCAA		
	FC: AAAGTGGCATTTCTCTACGC	213	Genotyping
	FT: AAAGTGGCATTTCTCTACGT		

rs685550 (C/T)	F: CCACATCAAACGTCGAGAGA	348	PCR
	R: TTGAATCTATGCGCTTGTCG		
	FC: ATATAGAGATATTAAACAGC	241	Genotyping
	FT: ATATAGAGATATTAAACAGT		

rs7520974 (A/G)	F: TCTCCAAATCAACACTCCTG	302	PCR
	R: CATGTCCTGTAGCACCATTT		
	FA: GCTGAAAGAAAGACAAATCA	175	Genotyping
	FG: GCTGAAAGAAAGACAAATCG		

rs3738435 (C/T)	F: CAGTGCATGCTACCAATTAT	361	PCR
	R: TGTAGCTGACGAAATGAGTG		
	FC: TAAAATAAGAGAATGAACGC	289	Genotyping
	FT: TAAAATAAGAGAATGAACGT		

CHRM3RT	F: ACCCAGCTCCGAGCAGATGGAC	339	RT-qPCR
	R: CGGCTGACTCTAGCTGGATGGG		
	Probe: TGCCTGAGGAGGAGCTGCTGGGGA TGGTGGACTTGGAGAG		

SNP: single-nucleotide polymorphism; F: forward; R: reverse.

**Table 3:** Genotype and allele frequencies of each marker analyzed in this study.

Locus		*N*	Allele^a^		*χ* ^2^	*p* value	Genotype^a^			*χ* ^2^	*p* value
			1	2			1/1	1/2	2/2		
rs2165870	P	30	50	10	0.056	0.813	22	6	2	0.108	0.947
	C	58	95	21			42	11	5		
rs10802789	P	30	39	21	0.006	0.938	12	15	3	0.879	0.644
	C	59	76	42			26	24	9		
rs685550	P	30	42	18	0.164	0.686	15	12	3	0.157	0.924
	C	59	86	32			32	22	5		
rs7520974	P	30	31	29	0.292	0.589	8	15	7	0.498	0.780
	C	59	66	52			20	26	13		
rs3738435	P	30	39	21	0.090	0.764	13	13	4	0.167	0.920
	C	59	74	44			23	28	8		

^a^1 indicates the major allele, and 2 indicates the minor allele.

P, patients with bladder cancer; C, control individuals.

**Table 4:** Haplotype frequency and test statistics between bladder cancer patients and controls.

Haplotype^a^	Numbers (frequency)			*p* value	OR (95% CI)	Permutation test
	Overall	Controls	Patients			*p*value^c^ [*W*, power]
G-T-T-G-T	34	25	9	0.251	0.614 (0.266–1.418)	0.317
G-T-T-A-C	33	27	6	0.025^*∗*^	0.350 (0.136–0.903)	0.027^*∗*^[0.17, 61%]
G-C-C-G-T	22	15	7	0.747	0.854 (0.328–2.225)	0.814
G-T-T-A-T	17	7	10	0.029^*∗*^	3.000 (1.079–8.344)	0.036^*∗*^[0.17, 61%]
G-C-C-A-C	12	5	7	0.077	2.826 (0.857–9.327)	0.112
A-C-T-G-T	8	8	0	0.034^*∗*^	—^b^	0.056 [0.16, 56%]
A-T-T-G-T	13	5	8	0.036^*∗*^	3.292 (1.027–10.560)	0.065 [0.14, 45%]
G-T-C-G-T	7	4	3	0.651	1.421 (0.307–6.569)	0.696
G-C-C-A-T	3	3	0	0.201	—^b^	0.549
G-T-T-G-C	6	3	3	0.429	1.912 (0.374–9.780)	0.430
G-C-T-G-T	6	4	2	0.935	0.931 (0.166–5.237)	1.000
A-T-T-A-C	4	4	0	0.139	—^b^	0.308
G-C-T-A-C	3	0	3	0.017^*∗*^	—^b^	0.044^*∗*^[0.18, 66%]
A-C-C-G-C	2	2	0	0.298	—^b^	0.542
A-C-T-A-C	1	0	1	0.171	—^b^	0.347
A-C-C-A-C	1	0	1	0.171	—^b^	0.350

Total *p* value: 1.946*E* − 3						
Global *p* value of permutation test^d^: 0.0008^*∗∗∗*^ [0.46, 99%]						

^*∗*^ *p* < 0.05 and ^*∗∗∗*^ *p* < 0.0001.

^a^Haplotypes were constructed with *CHRM3* SNPs: (from left to right) rs2165870, rs10802789, rs685550, rs7520974, and rs3738435.

^b^No OR was calculated for this haplotype.

^c^10000 permutations were replicated to evaluate permutation *p* value.

^d^The global *p* value was calculated using a permutation test (*n* = 10,000).
