# Supplementary material for: The Functional Haplotypes of CHRM3 Modulate mRNA Expression and Associate with Bladder Cancer among a Chinese Han Population in Kaohsiung City
Source: Biomed Res Int. 2016 Dec 7;2016:4052846. doi: 10.1155/2016/4052846 (PMC5174173; doi:10.1155/2016/4052846)

SUPPLEMENTARY DATA

Contains 1 table and 1 figure.

SUPPLEMENTARY TABLE

Supplementary Table1. Summary of plants and population on different areas in Taiwan, 2012.

Ranking	Area (City or County)	Number of plants in operation	Population
1	New Taipei City	18411	3939305
2	Taichung	15396	2684893
3	Taoyuan	10013	2030161
4	Tainan	8253	1881645
5	Changhua	8211	1299868
6	Kaohsiung	6306	2778659
7	Hsinchu	2897	949064
8	Chiayi	1991	804943
9	Miaoli	1629	563976
10	Yunlin	1517	710991
11	Taipei City	1158	2673226
12	Pingtung	952	858441
13	Yilan	907	458595
14	Hualien	367	335190
15	Keelung City	246	377153
16	Taitung	189	226252
17	Penghu	100	98843
18	Kinmen	81	113111
19	Lianjiang	4	11310

P.S : These data was downloaded from Environmental Protection Adminstration Executive Yuan, Taiwan (http://erdb.epa.gov.tw/ERDBIndex.aspx)

SUPPLEMENTARY FIGURE LEGEND

Figure S1. Sequencing results of CHRM3 SNPs. PCR products obtained with specific primer pairs were separated by agarose gel electrophoresis and stained with ethidium bromide. PCR products amplified from different genotype carriers were then sequencing by Sanger sequencing assay to further confirm the SNP calling results.

SUPPLEMENTARY FIGURE

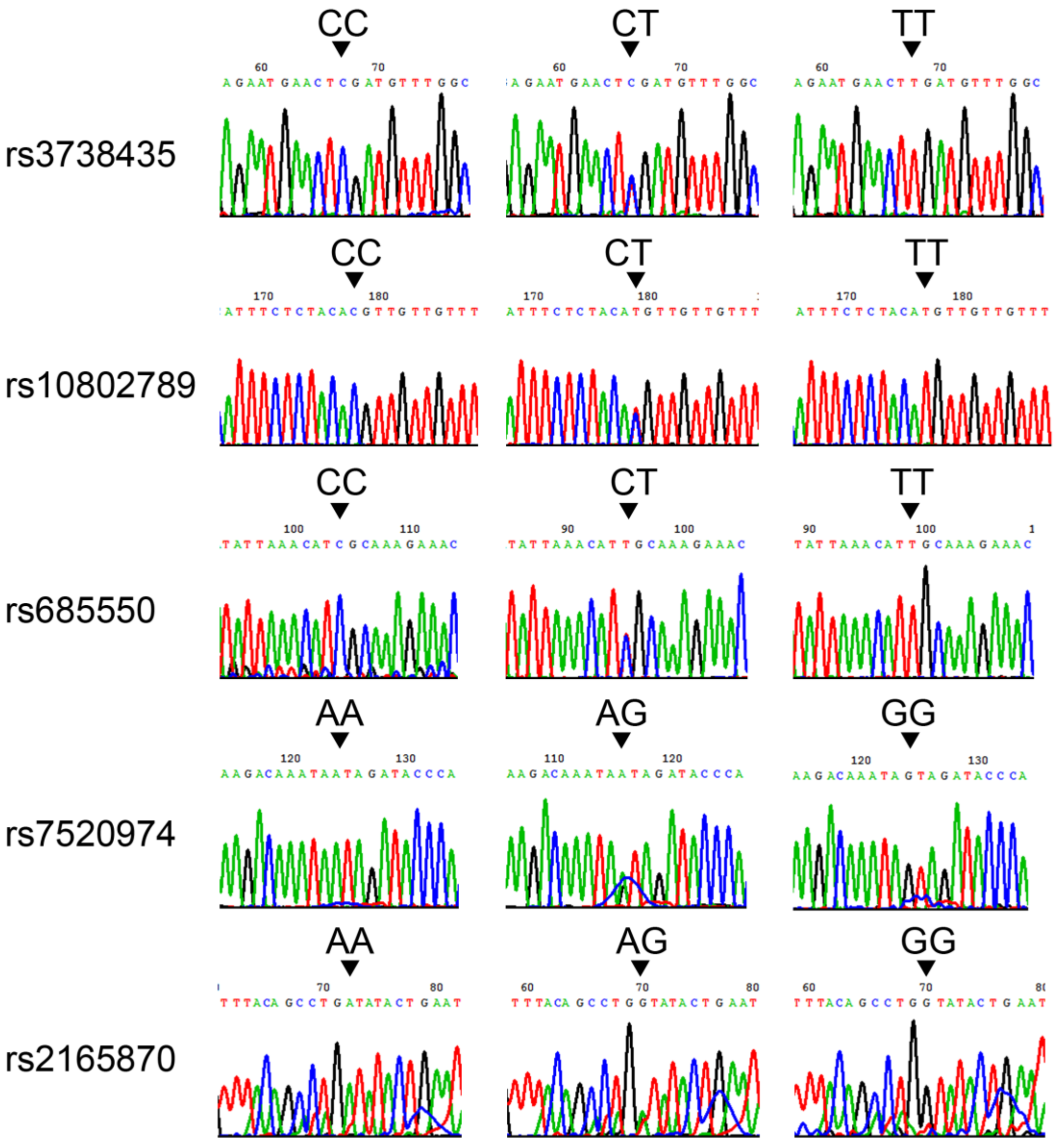

Supplement: Supplementary file 1 — The Supplementary Material included one supplementary table and one supplementary figure as follows. Supplementary Table1: Summary of plants and populations on different areas in Taiwan, 2012. Supplementary Figure S1: Sequencing results of CHRM3 SNPs. [file 4052846.f1.pdf]
